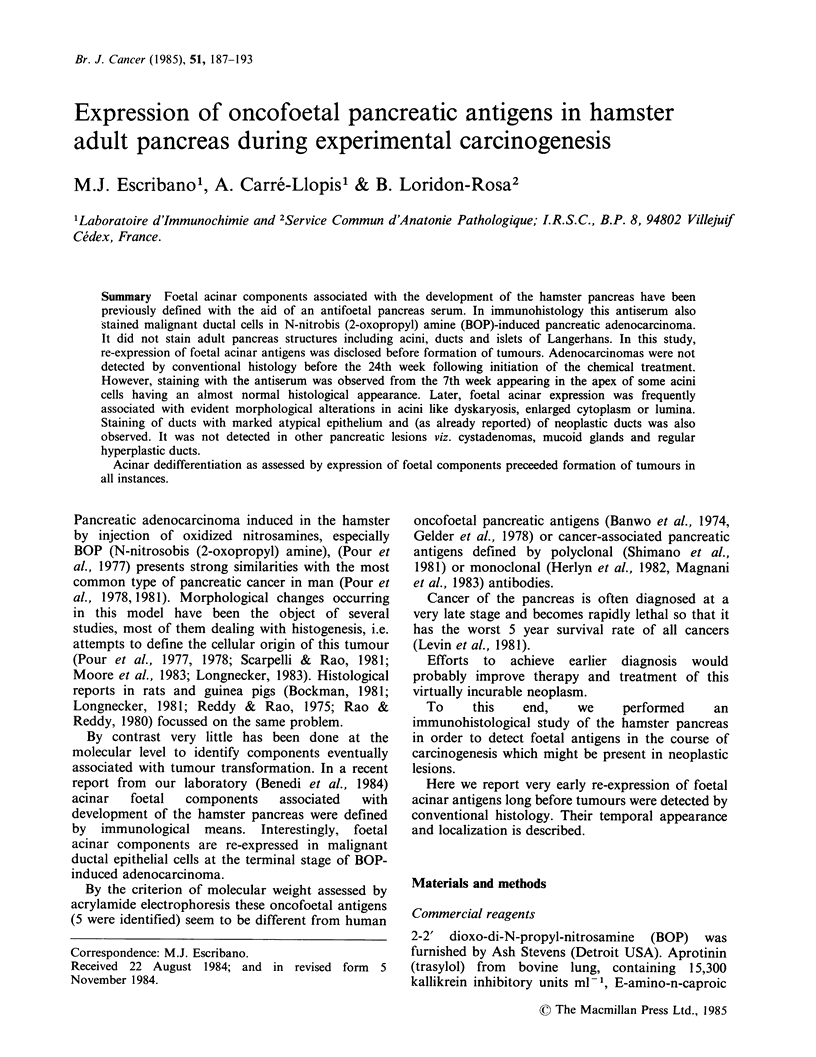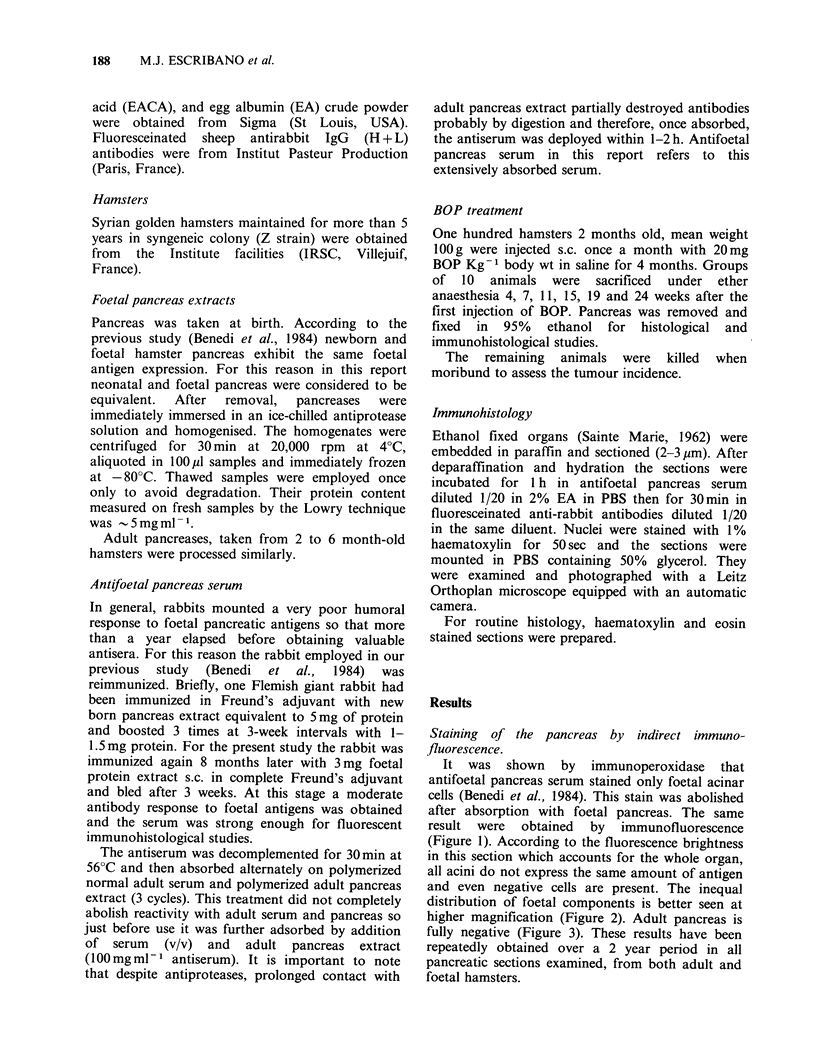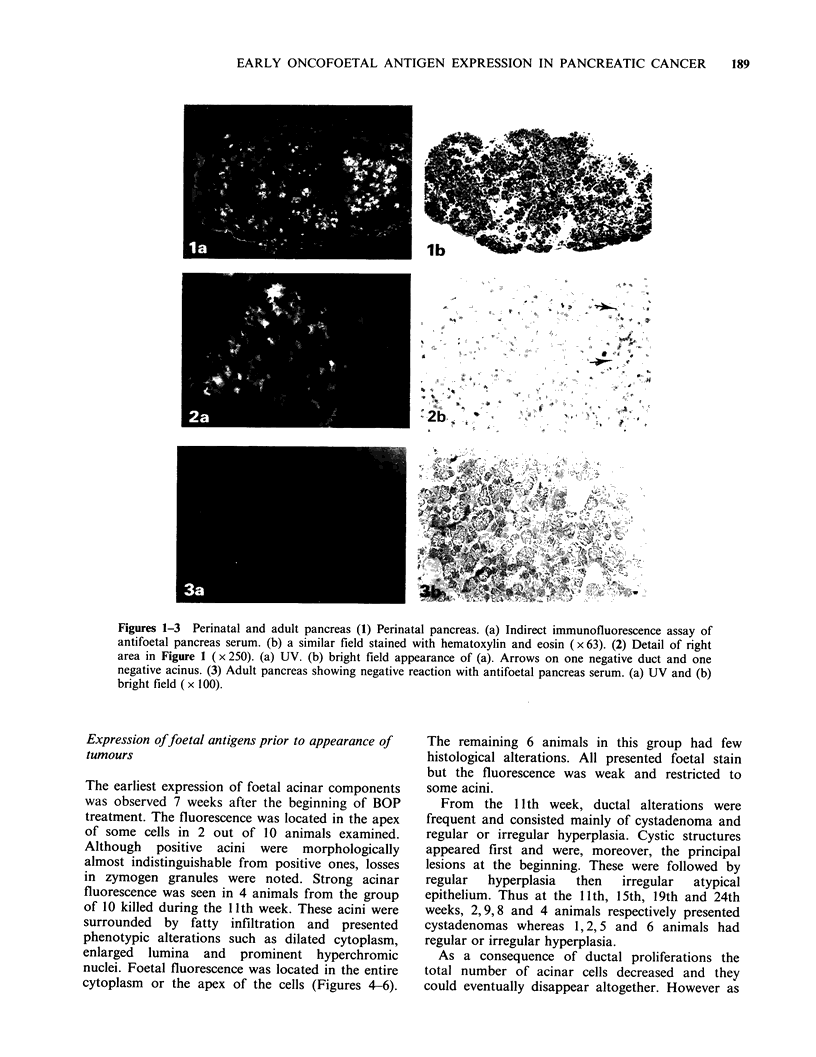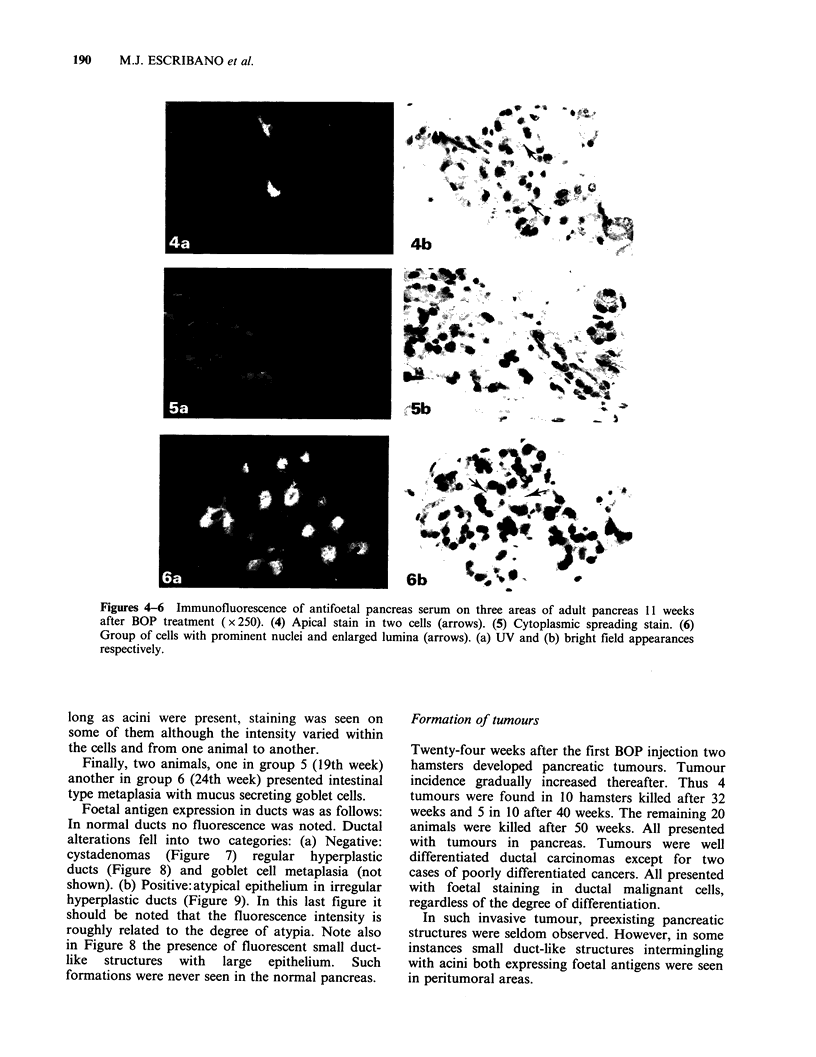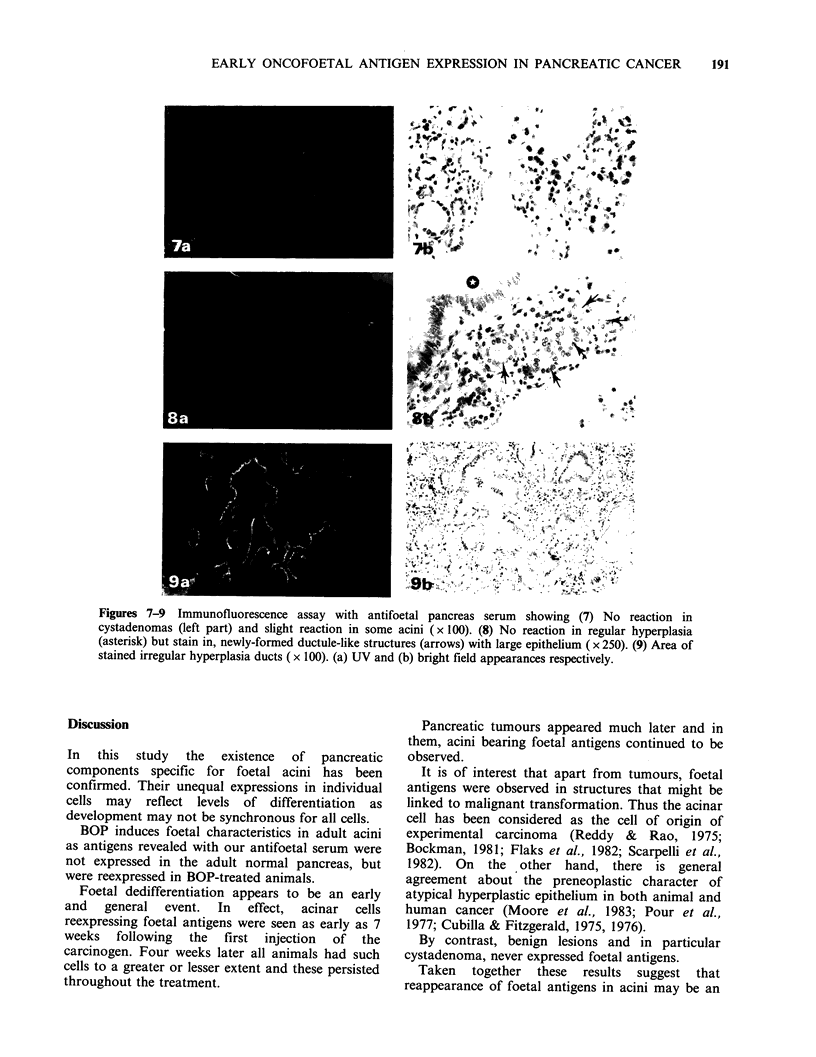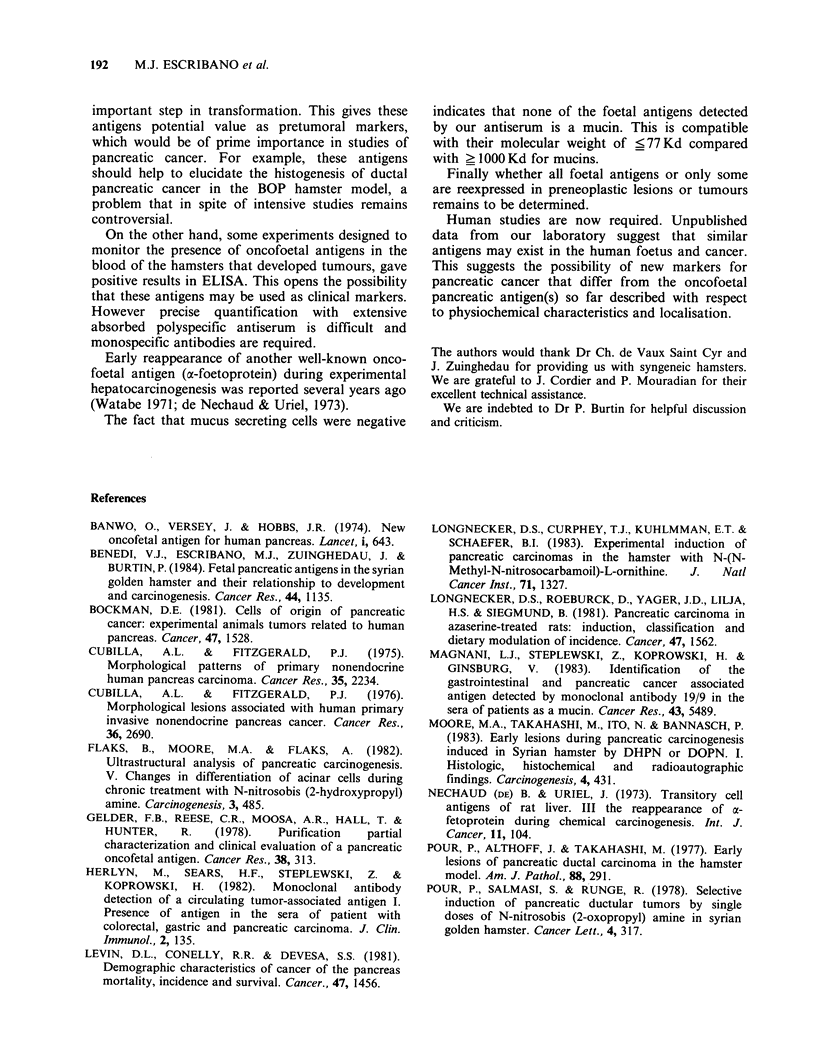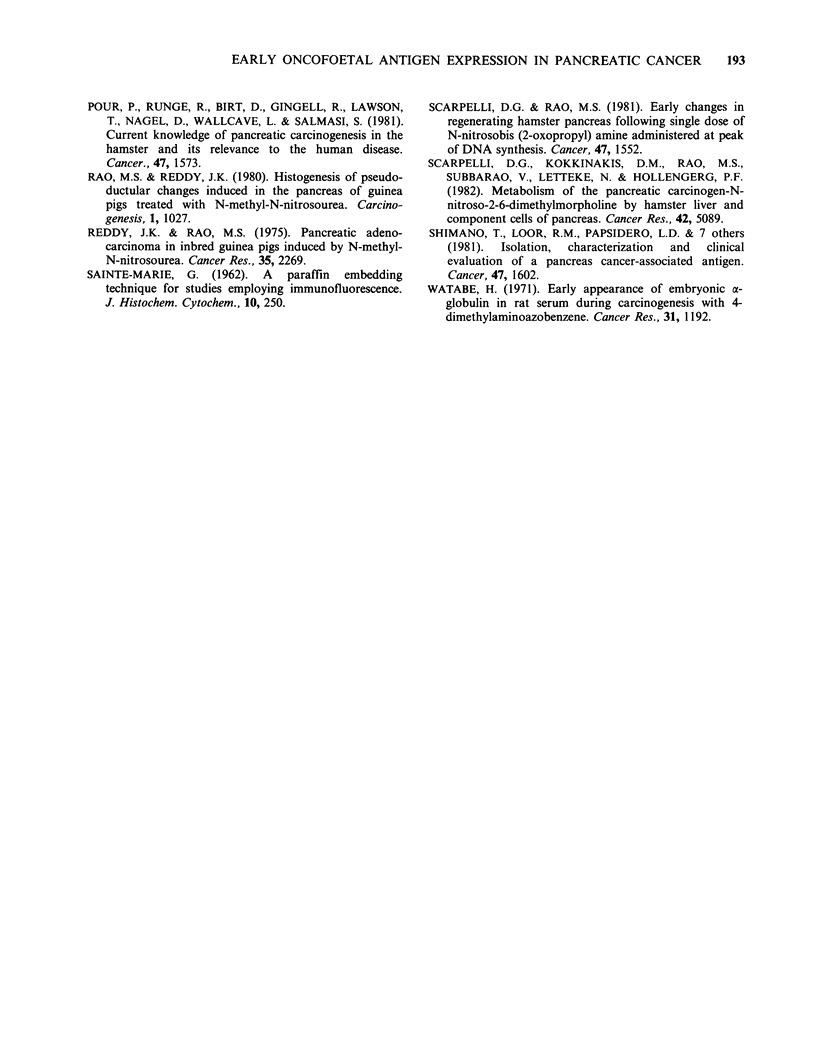# Expression of oncofoetal pancreatic antigens in hamster adult pancreas during experimental carcinogenesis.

**DOI:** 10.1038/bjc.1985.28

**Published:** 1985-02

**Authors:** M. J. Escribano, A. Carré-Llopis, B. Loridon-Rosa

## Abstract

**Images:**


*Br. J. Cancer* (1985), **51**, 187–193

### Expression of oncofoetal pancreatic antigens in hamster adult pancreas during experimental carcinogenesis

M.J. Escribano[1], A. Carré-Llopis[1] & B. Loridon-Rosa[2]

[1]*Laboratoire d'Immunochimie and* [2]*Service Commun d'Anatonie Pathologique; I.R.S.C., B.P. 8, 94802 Villejuif Cédex, France.*

**Summary** Foetal acinar components associated with the development of the hamster pancreas have been previously defined with the aid of an antifoetal pancreas serum. In immunohistology this antiserum also stained malignant ductal cells in N-nitrobis (2-oxopropyl) amine (BOP)-induced pancreatic adenocarcinoma. It did not stain adult pancreas structures including acini, ducts and islets of Langerhans. In this study, re-expression of foetal acinar antigens was disclosed before formation of tumours. Adenocarcinomas were not detected by conventional histology before the 24th week following initiation of the chemical treatment. However, staining with the antiserum was observed from the 7th week appearing in the apex of some acini cells having an almost normal histological appearance. Later, foetal acinar expression was frequently associated with evident morphological alterations in acini like dyskaryosis, enlarged cytoplasm or lumina. Staining of ducts with marked atypical epithelium and (as already reported) of neoplastic ducts was also observed. It was not detected in other pancreatic lesions *viz.* cystadenomas, mucoid glands and regular hyperplastic ducts.

Acinar dedifferentiation as assessed by expression of foetal components preceeded formation of tumours in all instances.

Pancreatic adenocarcinoma induced in the hamster by injection of oxidized nitrosamines, especially BOP (N-nitrosobis (2-oxopropyl) amine), (Pour *et al.*, 1977) presents strong similarities with the most common type of pancreatic cancer in man (Pour *et al.*, 1978, 1981). Morphological changes occurring in this model have been the object of several studies, most of them dealing with histogenesis, i.e. attempts to define the cellular origin of this tumour (Pour *et al.*, 1977, 1978; Scarpelli & Rao, 1981; Moore *et al.*, 1983; Longnecker, 1983). Histological reports in rats and guinea pigs (Bockman, 1981; Longnecker, 1981; Reddy & Rao, 1975; Rao & Reddy, 1980) focussed on the same problem.

By contrast very little has been done at the molecular level to identify components eventually associated with tumour transformation. In a recent report from our laboratory (Benedi *et al.*, 1984) acinar foetal components associated with development of the hamster pancreas were defined by immunological means. Interestingly, foetal acinar components are re-expressed in malignant ductal epithelial cells at the terminal stage of BOP-induced adenocarcinoma.

By the criterion of molecular weight assessed by acrylamide electrophoresis these oncofoetal antigens (5 were identified) seem to be different from human oncofoetal pancreatic antigens (Banwo *et al.*, 1974, Gelder *et al.*, 1978) or cancer-associated pancreatic antigens defined by polyclonal (Shimano *et al.*, 1981) or monoclonal (Herlyn *et al.*, 1982, Magnani *et al.*, 1983) antibodies.

Cancer of the pancreas is often diagnosed at a very late stage and becomes rapidly lethal so that it has the worst 5 year survival rate of all cancers (Levin *et al.*, 1981).

Efforts to achieve earlier diagnosis would probably improve therapy and treatment of this virtually incurable neoplasm.

To this end, we performed an immunohistological study of the hamster pancreas in order to detect foetal antigens in the course of carcinogenesis which might be present in neoplastic lesions.

Here we report very early re-expression of foetal acinar antigens long before tumours were detected by conventional histology. Their temporal appearance and localization is described.

### Materials and methods

### *Commercial reagents*

2-2′ dioxo-di-N-propyl-nitrosamine (BOP) was furnished by Ash Stevens (Detroit USA). Aprotinin (trasylol) from bovine lung, containing 15,300 kallikrein inhibitory units $ml^{-1}$, E-amino-n-caproic

Correspondence: M.J. Escribano.
Received 22 August 1984; and in revised form 5 November 1984.

© The Macmillan Press Ltd., 1985

acid (EACA), and egg albumin (EA) crude powder were obtained from Sigma (St Louis, USA). Fluoresceinated sheep antirabbit IgG (H+L) antibodies were from Institut Pasteur Production (Paris, France).

*Hamsters*

Syrian golden hamsters maintained for more than 5 years in syngeneic colony (Z strain) were obtained from the Institute facilities (IRSC, Villejuif, France).

*Foetal pancreas extracts*

Pancreas was taken at birth. According to the previous study (Benedi *et al.*, 1984) newborn and foetal hamster pancreas exhibit the same foetal antigen expression. For this reason in this report neonatal and foetal pancreas were considered to be equivalent. After removal, pancreases were immediately immersed in an ice-chilled antiprotease solution and homogenised. The homogenates were centrifuged for 30 min at 20,000 rpm at 4°C, aliquoted in 100 $\mu$l samples and immediately frozen at −80°C. Thawed samples were employed once only to avoid degradation. Their protein content measured on fresh samples by the Lowry technique was $\sim 5\,mg\,ml^{-1}$.

Adult pancreases, taken from 2 to 6 month-old hamsters were processed similarly.

*Antifoetal pancreas serum*

In general, rabbits mounted a very poor humoral response to foetal pancreatic antigens so that more than a year elapsed before obtaining valuable antisera. For this reason the rabbit employed in our previous study (Benedi *et al.*, 1984) was reimmunized. Briefly, one Flemish giant rabbit had been immunized in Freund's adjuvant with new born pancreas extract equivalent to 5 mg of protein and boosted 3 times at 3-week intervals with 1–1.5 mg protein. For the present study the rabbit was immunized again 8 months later with 3 mg foetal protein extract s.c. in complete Freund's adjuvant and bled after 3 weeks. At this stage a moderate antibody response to foetal antigens was obtained and the serum was strong enough for fluorescent immunohistological studies.

The antiserum was decomplemented for 30 min at 56°C and then absorbed alternately on polymerized normal adult serum and polymerized adult pancreas extract (3 cycles). This treatment did not completely abolish reactivity with adult serum and pancreas so just before use it was further adsorbed by addition of serum (v/v) and adult pancreas extract ($100\,mg\,ml^{-1}$ antiserum). It is important to note that despite antiproteases, prolonged contact with adult pancreas extract partially destroyed antibodies probably by digestion and therefore, once absorbed, the antiserum was deployed within 1–2 h. Antifoetal pancreas serum in this report refers to this extensively absorbed serum.

*BOP treatment*

One hundred hamsters 2 months old, mean weight 100 g were injected s.c. once a month with 20 mg BOP $Kg^{-1}$ body wt in saline for 4 months. Groups of 10 animals were sacrificed under ether anaesthesia 4, 7, 11, 15, 19 and 24 weeks after the first injection of BOP. Pancreas was removed and fixed in 95% ethanol for histological and immunohistological studies.

The remaining animals were killed when moribund to assess the tumour incidence.

*Immunohistology*

Ethanol fixed organs (Sainte Marie, 1962) were embedded in paraffin and sectioned (2–3 $\mu$m). After deparaffination and hydration the sections were incubated for 1 h in antifoetal pancreas serum diluted 1/20 in 2% EA in PBS then for 30 min in fluoresceinated anti-rabbit antibodies diluted 1/20 in the same diluent. Nuclei were stained with 1% haematoxylin for 50 sec and the sections were mounted in PBS containing 50% glycerol. They were examined and photographed with a Leitz Orthoplan microscope equipped with an automatic camera.

For routine histology, haematoxylin and eosin stained sections were prepared.

### Results

*Staining of the pancreas by indirect immunofluorescence.*

It was shown by immunoperoxidase that antifoetal pancreas serum stained only foetal acinar cells (Benedi *et al.*, 1984). This stain was abolished after absorption with foetal pancreas. The same result were obtained by immunofluorescence (Figure 1). According to the fluorescence brightness in this section which accounts for the whole organ, all acini do not express the same amount of antigen and even negative cells are present. The inequal distribution of foetal components is better seen at higher magnification (Figure 2). Adult pancreas is fully negative (Figure 3). These results have been repeatedly obtained over a 2 year period in all pancreatic sections examined, from both adult and foetal hamsters.

**Figures 1–3** Perinatal and adult pancreas (**1**) Perinatal pancreas. (a) Indirect immunofluorescence assay of antifoetal pancreas serum. (b) a similar field stained with hematoxylin and eosin (×63). (**2**) Detail of right area in **Figure 1** (×250). (a) UV. (b) bright field appearance of (a). Arrows on one negative duct and one negative acinus. (**3**) Adult pancreas showing negative reaction with antifoetal pancreas serum. (a) UV and (b) bright field (×100).

*Expression of foetal antigens prior to appearance of tumours*

The earliest expression of foetal acinar components was observed 7 weeks after the beginning of BOP treatment. The fluorescence was located in the apex of some cells in 2 out of 10 animals examined. Although positive acini were morphologically almost indistinguishable from positive ones, losses in zymogen granules were noted. Strong acinar fluorescence was seen in 4 animals from the group of 10 killed during the 11th week. These acini were surrounded by fatty infiltration and presented phenotypic alterations such as dilated cytoplasm, enlarged lumina and prominent hyperchromic nuclei. Foetal fluorescence was located in the entire cytoplasm or the apex of the cells (Figures 4–6).

The remaining 6 animals in this group had few histological alterations. All presented foetal stain but the fluorescence was weak and restricted to some acini.

From the 11th week, ductal alterations were frequent and consisted mainly of cystadenoma and regular or irregular hyperplasia. Cystic structures appeared first and were, moreover, the principal lesions at the beginning. These were followed by regular hyperplasia then irregular atypical epithelium. Thus at the 11th, 15th, 19th and 24th weeks, 2, 9, 8 and 4 animals respectively presented cystadenomas whereas 1, 2, 5 and 6 animals had regular or irregular hyperplasia.

As a consequence of ductal proliferations the total number of acinar cells decreased and they could eventually disappear altogether. However as

**Figures 4–6** Immunofluorescence of antifoetal pancreas serum on three areas of adult pancreas 11 weeks after BOP treatment ( × 250). (**4**) Apical stain in two cells (arrows). (**5**) Cytoplasmic spreading stain. (**6**) Group of cells with prominent nuclei and enlarged lumina (arrows). (a) UV and (b) bright field appearances respectively.

long as acini were present, staining was seen on some of them although the intensity varied within the cells and from one animal to another.

Finally, two animals, one in group 5 (19th week) another in group 6 (24th week) presented intestinal type metaplasia with mucus secreting goblet cells.

Foetal antigen expression in ducts was as follows: In normal ducts no fluorescence was noted. Ductal alterations fell into two categories: (a) Negative: cystadenomas (Figure 7) regular hyperplastic ducts (Figure 8) and goblet cell metaplasia (not shown). (b) Positive: atypical epithelium in irregular hyperplastic ducts (Figure 9). In this last figure it should be noted that the fluorescence intensity is roughly related to the degree of atypia. Note also in Figure 8 the presence of fluorescent small duct-like structures with large epithelium. Such formations were never seen in the normal pancreas.

*Formation of tumours*

Twenty-four weeks after the first BOP injection two hamsters developed pancreatic tumours. Tumour incidence gradually increased thereafter. Thus 4 tumours were found in 10 hamsters killed after 32 weeks and 5 in 10 after 40 weeks. The remaining 20 animals were killed after 50 weeks. All presented with tumours in pancreas. Tumours were well differentiated ductal carcinomas except for two cases of poorly differentiated cancers. All presented with foetal staining in ductal malignant cells, regardless of the degree of differentiation.

In such invasive tumour, preexisting pancreatic structures were seldom observed. However, in some instances small duct-like structures intermingling with acini both expressing foetal antigens were seen in peritumoral areas.

**Figures 7–9** Immunofluorescence assay with antifoetal pancreas serum showing (7) No reaction in cystadenomas (left part) and slight reaction in some acini (×100). (8) No reaction in regular hyperplasia (asterisk) but stain in, newly-formed ductule-like structures (arrows) with large epithelium (×250). (9) Area of stained irregular hyperplasia ducts (×100). (a) UV and (b) bright field appearances respectively.

### Discussion

In this study the existence of pancreatic components specific for foetal acini has been confirmed. Their unequal expressions in individual cells may reflect levels of differentiation as development may not be synchronous for all cells.

BOP induces foetal characteristics in adult acini as antigens revealed with our antifoetal serum were not expressed in the adult normal pancreas, but were reexpressed in BOP-treated animals.

Foetal dedifferentiation appears to be an early and general event. In effect, acinar cells reexpressing foetal antigens were seen as early as 7 weeks following the first injection of the carcinogen. Four weeks later all animals had such cells to a greater or lesser extent and these persisted throughout the treatment.

Pancreatic tumours appeared much later and in them, acini bearing foetal antigens continued to be observed.

It is of interest that apart from tumours, foetal antigens were observed in structures that might be linked to malignant transformation. Thus the acinar cell has been considered as the cell of origin of experimental carcinoma (Reddy & Rao, 1975; Bockman, 1981; Flaks *et al.*, 1982; Scarpelli *et al.*, 1982). On the other hand, there is general agreement about the preneoplastic character of atypical hyperplastic epithelium in both animal and human cancer (Moore *et al.*, 1983; Pour *et al.*, 1977; Cubilla & Fitzgerald, 1975, 1976).

By contrast, benign lesions and in particular cystadenoma, never expressed foetal antigens.

Taken together these results suggest that reappearance of foetal antigens in acini may be an important step in transformation. This gives these antigens potential value as pretumoral markers, which would be of prime importance in studies of pancreatic cancer. For example, these antigens should help to elucidate the histogenesis of ductal pancreatic cancer in the BOP hamster model, a problem that in spite of intensive studies remains controversial.

On the other hand, some experiments designed to monitor the presence of oncofoetal antigens in the blood of the hamsters that developed tumours, gave positive results in ELISA. This opens the possibility that these antigens may be used as clinical markers. However precise quantification with extensive absorbed polyspecific antiserum is difficult and monospecific antibodies are required.

Early reappearance of another well-known oncofoetal antigen (α-foetoprotein) during experimental hepatocarcinogenesis was reported several years ago (Watabe 1971; de Nechaud & Uriel, 1973).

The fact that mucus secreting cells were negative indicates that none of the foetal antigens detected by our antiserum is a mucin. This is compatible with their molecular weight of $\leqq 77$ Kd compared with $\geqq 1000$ Kd for mucins.

Finally whether all foetal antigens or only some are reexpressed in preneoplastic lesions or tumours remains to be determined.

Human studies are now required. Unpublished data from our laboratory suggest that similar antigens may exist in the human foetus and cancer. This suggests the possibility of new markers for pancreatic cancer that differ from the oncofoetal pancreatic antigen(s) so far described with respect to physiochemical characteristics and localisation.

The authors would thank Dr Ch. de Vaux Saint Cyr and J. Zuinghedau for providing us with syngeneic hamsters. We are grateful to J. Cordier and P. Mouradian for their excellent technical assistance.

We are indebted to Dr P. Burtin for helpful discussion and criticism.

### References

BANWO, O., VERSEY, J. & HOBBS, J.R. (1974). New oncofetal antigen for human pancreas. *Lancet*, **i**, 643.

BENEDI, V.J., ESCRIBANO, M.J., ZUINGHEDAU, J. & BURTIN, P. (1984). Fetal pancreatic antigens in the syrian golden hamster and their relationship to development and carcinogenesis. *Cancer Res.*, **44**, 1135.

BOCKMAN, D.E. (1981). Cells of origin of pancreatic cancer: experimental animals tumors related to human pancreas. *Cancer*, **47**, 1528.

CUBILLA, A.L. & FITZGERALD, P.J. (1975). Morphological patterns of primary nonendocrine human pancreas carcinoma. *Cancer Res.*, **35**, 2234.

CUBILLA, A.L. & FITZGERALD, P.J. (1976). Morphological lesions associated with human primary invasive nonendocrine pancreas cancer. *Cancer Res.*, **36**, 2690.

FLAKS, B., MOORE, M.A. & FLAKS, A. (1982). Ultrastructural analysis of pancreatic carcinogenesis. V. Changes in differentiation of acinar cells during chronic treatment with N-nitrosobis (2-hydroxypropyl) amine. *Carcinogenesis*, **3**, 485.

GELDER, F.B., REESE, C.R., MOOSA, A.R., HALL, T. & HUNTER, R. (1978). Purification partial characterization and clinical evaluation of a pancreatic oncofetal antigen. *Cancer Res.*, **38**, 313.

HERLYN, M., SEARS, H.F., STEPLEWSKI, Z. & KOPROWSKI, H. (1982). Monoclonal antibody detection of a circulating tumor-associated antigen I. Presence of antigen in the sera of patient with colorectal, gastric and pancreatic carcinoma. *J. Clin. Immunol.*, **2**, 135.

LEVIN, D.L., CONELLY, R.R. & DEVESA, S.S. (1981). Demographic characteristics of cancer of the pancreas mortality, incidence and survival. *Cancer.*, **47**, 1456.

LONGNECKER, D.S., CURPHEY, T.J., KUHLMMAN, E.T. & SCHAEFER, B.I. (1983). Experimental induction of pancreatic carcinomas in the hamster with N-(N-Methyl-N-nitrosocarbamoil)-L-ornithine. *J. Natl Cancer Inst.*, **71**, 1327.

LONGNECKER, D.S., ROEBURCK, D., YAGER, J.D., LILJA, H.S. & SIEGMUND, B. (1981). Pancreatic carcinoma in azaserine-treated rats: induction, classification and dietary modulation of incidence. *Cancer*, **47**, 1562.

MAGNANI, L.J., STEPLEWSKI, Z., KOPROWSKI, H. & GINSBURG, V. (1983). Identification of the gastrointestinal and pancreatic cancer associated antigen detected by monoclonal antibody 19/9 in the sera of patients as a mucin. *Cancer Res.*, **43**, 5489.

MOORE, M.A., TAKAHASHI, M., ITO, N. & BANNASCH, P. (1983). Early lesions during pancreatic carcinogenesis induced in Syrian hamster by DHPN or DOPN. I. Histologic, histochemical and radioautographic findings. *Carcinogenesis*, **4**, 431.

NECHAUD (DE) B. & URIEL, J. (1973). Transitory cell antigens of rat liver. III the reappearance of α-fetoprotein during chemical carcinogenesis. *Int. J. Cancer*, **11**, 104.

POUR, P., ALTHOFF, J. & TAKAHASHI, M. (1977). Early lesions of pancreatic ductal carcinoma in the hamster model. *Am. J. Pathol.*, **88**, 291.

POUR, P., SALMASI, S. & RUNGE, R. (1978). Selective induction of pancreatic ductular tumors by single doses of N-nitrosobis (2-oxopropyl) amine in syrian golden hamster. *Cancer Lett.*, **4**, 317.

POUR, P., RUNGE, R., BIRT, D., GINGELL, R., LAWSON, T., NAGEL, D., WALLCAVE, L. & SALMASI, S. (1981). Current knowledge of pancreatic carcinogenesis in the hamster and its relevance to the human disease. *Cancer.*, **47,** 1573.

RAO, M.S. & REDDY, J.K. (1980). Histogenesis of pseudo-ductular changes induced in the pancreas of guinea pigs treated with N-methyl-N-nitrosourea. *Carcinogenesis,* **1,** 1027.

REDDY, J.K. & RAO, M.S. (1975). Pancreatic adenocarcinoma in inbred guinea pigs induced by N-methyl-N-nitrosourea. *Cancer Res.*, **35,** 2269.

SAINTE-MARIE, G. (1962). A paraffin embedding technique for studies employing immunofluorescence. *J. Histochem. Cytochem.*, **10,** 250.

SCARPELLI, D.G. & RAO, M.S. (1981). Early changes in regenerating hamster pancreas following single dose of N-nitrosobis (2-oxopropyl) amine administered at peak of DNA synthesis. *Cancer,* **47,** 1552.

SCARPELLI, D.G., KOKKINAKIS, D.M., RAO, M.S., SUBBARAO, V., LETTEKE, N. & HOLLENGERG, P.F. (1982). Metabolism of the pancreatic carcinogen-N-nitroso-2-6-dimethylmorpholine by hamster liver and component cells of pancreas. *Cancer Res.*, **42,** 5089.

SHIMANO, T., LOOR, R.M., PAPSIDERO, L.D. & 7 others (1981). Isolation, characterization and clinical evaluation of a pancreas cancer-associated antigen. *Cancer,* **47,** 1602.

WATABE, H. (1971). Early appearance of embryonic α-globulin in rat serum during carcinogenesis with 4-dimethylaminoazobenzene. *Cancer Res.*, **31,** 1192.